# Insights into Hydration Kinetics of Cement Pastes Evaluated by Low-Field Nuclear Magnetic Resonance: Effects of Super-Absorbent Polymer as Internal Curing Agent and Calcium Oxide as Expansive Agent

**DOI:** 10.3390/ma18040836

**Published:** 2025-02-14

**Authors:** Meixin Liu, Yuan Hu, Jing Li, Xiaolin Liu, Huiwen Sun, Yunfei Di, Xia Wu, Junyi Zhang

**Affiliations:** 1 College of Civil and Transportation Engineering, Hohai University, Nanjing 210098, China; liumeix1999@163.com (M.L.); 18705609882@163.com (X.L.);; 2 The Huaihe River Commission of the Ministry of Water Resources, Bengbu 233001, China

**Keywords:** cement paste, hydration kinetics, super-absorbent polymer (SAP), CaO-based expansive admixture, low-field nuclear magnetic resonance (LF-NMR)

## Abstract

Understanding the hydration kinetics of cement paste is essential for adjusting the early-age performance of concrete. Low-field nuclear magnetic resonance (LF-NMR) has emerged as an innovative technique to evaluate cement hydration progress by analyzing the evolution of transverse relaxation time (*T*_2_) signals. This study provides insights into the influence of a super-absorbent polymer (SAP) as an internal curing agent and calcium oxide (CaO) as an expansive agent (EA) on LF-NMR spectroscopy of cement paste for the first time. The chemical compositions of the cement and CaO-based EA were determined by X-ray fluorescence, while the morphological characterizations of the cement, SAP and CaO-based EA materials were characterized by scanning electron microscopy. Based on the extreme points in the first-order derivatives of the *T*_2_ signal maximum amplitude curve, the hydration progress was analyzed and identified with four stages in detail. The results showed that the use of the SAP with a higher content retarded the hydration kinetics more evidently at a very early age, thus prolonging the duration of the induction and acceleration stages. The use of the CaO-based EA shortened the induction, acceleration and deceleration stages, which verified its promotion of hydration kinetics in the presence of the SAP. The combination of 3 wt% SAP and 2 wt% CaO consumed more water content synergistically in the first 100 h by hydration reactions. These findings revealed the roles of SAP and CaO-based EA (commonly adopted for low-shrinkage concrete) in adjusting hydration parameters and the microstructure evolution of cement-based materials, which would further offer fundamental knowledge for the early-age cracking control of concrete structures.

## 1. Introduction

The generation of cracks in concrete can provide permeable invasion pathways for corrosive media and then induce subsequent propagation and further expansion of the cracks, which will aggravate the performance deterioration of a concrete structure [1,2]. The cracking of concrete at an early age could be one of the most common problems in practical concrete engineering, and volume deformation is one of the key factors that lead to early-age cracks [3,4]. Incorporating an expansive admixture can compensate for the shrinkage volumetric deformation of concrete, which is regarded as an effective approach to mitigating shrinkage-induced cracking [5,6]. Meanwhile, an internal curing admixture is favorable for reserving the internal humidity of concrete and helps to prevent cracks caused by autogenous shrinkage [7,8,9]. Specifically, calcium oxide (CaO) has been proven to possess a fast expansion function and extensive adaptability in different environmental conditions [10]. In addition, the use of a super-absorbent polymer (SAP) can alleviate the autogenous shrinkage effectively and enhance the long-term hydration degree of concrete, which has little effect on the mechanical strength of concrete compared with other internal curing materials [11,12]. Hence, these two chemical admixtures are often incorporated simultaneously for the shrinkage and cracking control of concrete [13,14,15].

Understanding the hydration kinetics of concretes with CaO or SAP can be crucial for tailoring their fresh and hardened properties. Wczelik et al. [16] investigated the hydration kinetics of cement-based materials by isothermal calorimetry and reported that the first exothermic peak was heightened and broadened with the addition of an expansive additive, while the second exothermic peak was lowered. Zhao et al. [17] found that the expansion additive could shorten the setting and hardening of fresh cement-based materials. Wang et al. [18] used quantitative X-ray diffraction (QXRD) to investigate the influence of SAP incorporation on cement hydration and assess the hydration degree and found that an SAP as an internal curing admixture slightly decreased the hydration degree at an early age. An environmental advanced scanning electron microscope (ESEM) was also utilized to observe the absorption behavior of SAP particles in a cement pore solution and their shape variations, which could facilitate understanding the mechanism for SAPs restraining concrete autogenous shrinkage during the specific hydration progress of cement [19].

On the other hand, the hydration kinetics of cementitious binders have a direct effect on the physical and mechanical properties of early-age concrete. Some conventional techniques have been adopted to characterize hydration, such as isothermal calorimetry, electrical resistivity measurement, scanning electron microscopy (SEM) and transmission electron microscopy (TEM) [16,20,21,22]. To be specific, isothermal calorimetry has been commonly employed for the evaluation of hydration kinetics, but at the later stage, its signal usually becomes too weak after hydration for several days. Electrical impedance spectroscopy was also developed as a non-contact measurement technique for hydration characterization. Nevertheless, analyzing the alternating electric current would be complicated, and the frequency variation also affects resistivity. Furthermore, it is difficult to realize the continuous monitoring of hydration via SEM and TEM observation approaches, and the pre-drying treatment will also change the microstructure of cement samples.

Therefore, low-field nuclear magnetic resonance (LF-NMR), as a novel approach, was proposed to investigate hydration kinetics and the microstructure evolution of a cement paste by assessing the relaxation time of hydrogen protons in pores [23,24]. Considering that LF-NMR is a type of non-destructive, non-invasive and in situ continuous characterization, it shows significant advantages compared with other conventional measurement approaches. Wang et al. [25] used an LF-NMR spectrometer to characterize the hydration kinetics of cement paste and pointed out that the hydration periods could be clearly described by the evolution of the longitudinal relaxation time (*T*_1_) signal. Similarly, monitoring the intensity of the transverse relaxation time (*T*_2_) signal of LF-NMR of the cement paste could also characterize the hydration kinetics, which presented a good agreement with other evaluation approaches [26]. In addition, the measurement of the *T*_2_ signal required much shorter time and possessed higher robustness than that of the *T*_1_ signal, by which the hydration mechanism could be revealed according to the time-dependent curve of the *T*_2_ signal [27].

According to the cement chemistry theory, the multi-stage hydration progress could be analyzed and identified by establishing a hydration model, which can be adopted to quantify the hydration kinetics as well as divide several specific hydration periods for cement-based materials [28,29]. The determination of the dynamic hydration model can unravel the relation between hydration kinetics and various influential factors. However, little research has focused on the coupled effects of an SAP and CaO on cement hydration for shrinkage control, particularly using an advanced characterization technique like LF-NMR. This research gap shed light on the urgent need and significance for a comprehensive understanding of how these chemical admixtures interact to change the hydration kinetics and microstructure evolution of cement-based materials.

Since there is still little research focusing on the hydration characteristics of cement-based materials with CaO as the expansive admixture (EA) and an SAP as the internal curing admixture concurrently, the influences of the water-to-cement ratio (*w*/*c*), SAP and CaO-based EA on essential parameters of hydration models were analyzed for the first time in this research. Specifically, the effects of the SAP and/or CaO-based EA incorporation on durations of hydration stages (including the induction, acceleration, deceleration and stable stages) were investigated based on the second-order differential functions of *T*_2_ signal curves. The findings are expected to provide fundamental knowledge for optimizing the design of low-shrinkage concrete.

## 2. Materials and Methods

### 2.1. Materials

Portland cement with a strength grade of 52.5 (P·I 52.5) in compliance with Chinese Standard GB 175-2023 [30] was chosen in this study, which possessed a specific surface area of 373.2 m^2^/kg. A commercially available concrete expansive admixture (EA) with CaO as the active component was used, which had a restrained expansion ratio of 0.18% after water curing for 7 days (according to Chinese Standard GB/T 23439-2017) [31]. A super-absorbent polymer (SAP) with a particle size of 75–95 μm was also employed as the internal curing admixture, which presented a spherical shape and possessed a water absorption capacity of 19.7 g per gram according to the tea bag method. The tea bag method was adopted to measure the absorption capacity of the SAP as (m3 − m2 − m0)/(m2 − m1), where m1 is the mass of an empty tea bag, m2 is the mass after loading with the SAP, m3 is the swollen mass after immersion in the test solution and m0 is the average liquid absorption mass of ten tea bags.

### 2.2. Methods

#### 2.2.1. Sample Preparation

The water-to-cement ratio (*w*/*c*) was designed at 0.30, 0.35 or 0.40 for the cement paste mixture. For keeping consistent with practical cases, the dosage of the SAP was 0.15 wt% or 0.30 wt% (by mass of cement), while the dosage of the CaO-based EA was 2.0 wt% or 3.0 wt% (by mass for the replacement of cement). The mixing proportions of all samples are provided in Table 1, in which the “CP” series represents blank samples, while the “CP_S” series denotes samples containing the SAP.

The stirring and homogenization processes are as follows. First, the cement, EA and SAP solids were dry-mixed using a mechanical mixer for 2 min to ensure a uniform distribution of the dry materials. Then, mixing water was added to the container and further mixed with the low speed of 140 rev/min for 60 s. Afterwards, the mixing speed was increased to 285 rev/min for another 150 s, which led to the adequate dispersion state of the fresh cement paste.

#### 2.2.2. Morphology Analysis

The microscopic morphologies of the cement, the SAP and the CaO-based EA were examined using scanning electron microscopy (SEM) (Sigma300, ZEISS, Oberkochen, Germany). Nonconductive samples were coated with a 30 s gold film to improve conductivity.

#### 2.2.3. Chemical Composition Analysis

The chemical composition of the cement and CaO-based EA was determined using an X-ray fluorescence spectrum analyzer (ZSX primus II, Rigaku, Tokyo, Japan). Powder samples of about 3 g were dried and passed through a 200-mesh sieve and then exposed to X-rays to emit fluorescence for data collection.

#### 2.2.4. Hydration Characteristics Analysis

**Figure 1:**
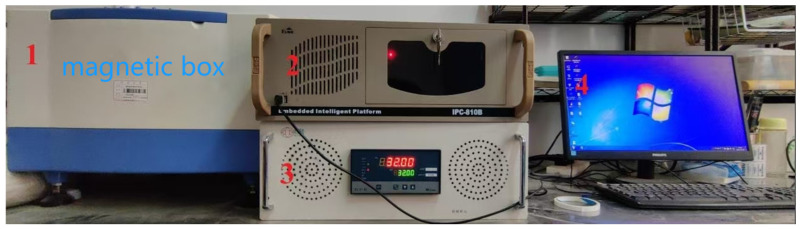
LF-NMR instrument: (1) magnetic box; (2) spectrum system and controlling machine; (3) console unit; (4) displayer.

The experiment utilized an LF-NMR instrument (PQ-001, Niumag, Suzhou, China), as shown in Figure 1. This device is equipped with a low magnetic field with a field strength of only 0.42 T. The frequency was set at 18 MHz. The temperature of the magnet was controlled at 32 ± 0.02 °C, and the laboratory temperature was maintained at 20 ± 2 °C. The measurement of the transverse relaxation time *T*_2_ was carried out using the Carr–Purcell–Meiboom–Gill (CPMG) method.

### 2.3. Hydration Kinetic Characterizations

Fresh mixture was filled into the glass tube to evaluate the hydration kinetics immediately based on the LF-NMR spectroscopy, with a magnetic field intensity of 0.42 T and a proton resonance frequency of 18 MHz. Then, the sample together with the tube was stored in an incubator with a constant temperature of 20 °C during the testing intervals. The detailed calibration process and parameter information for the LF-NMR measurement were consistent with the previous studies [23,24], and the transverse relaxation (*T*_2_) signal of the cement paste sample was calculated by the Carr–Purcell–Meiboom–Gill (CPMG) sequence analysis.

**Figure 2:**
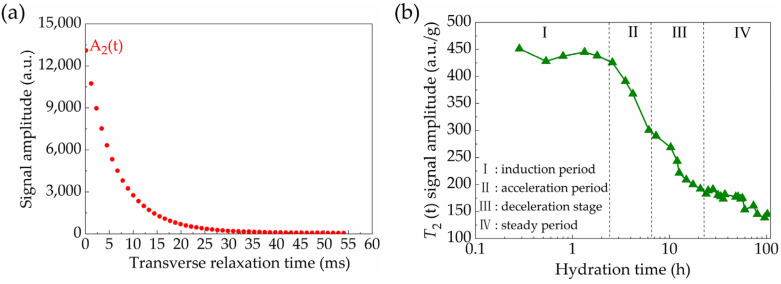
(**a**) First wave of *T*_2_ signal; (**b**) time-dependent evolution of *T*_2_ signal maximum amplitude by unit mass of sample.

**Figure 3:**
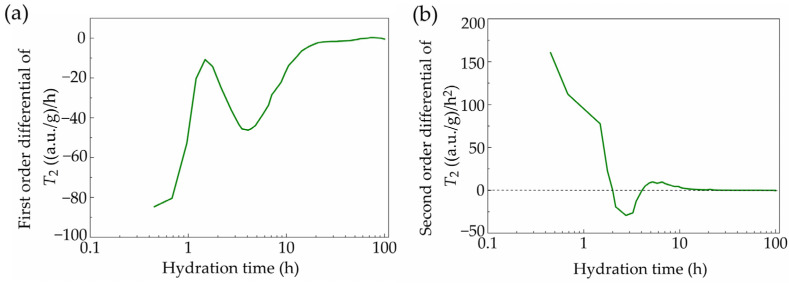
(**a**) First-order and (**b**) second-order differential of *T*_2_ signal maximum amplitude.

For example, the amplitude of the *T*_2_ signal by a single transient measurement is shown in Figure 2a, in which the maximum value could indicate the free water amount in the sample. The signal intensity of the physically bound water is expressed by absorbance units (a.u.s). Then, the time-dependent evolution curve of the maximum *T*_2_ amplitude could be plotted according to the long-term continuous measurements, as shown in Figure 2b. Based on the first-order (and further second-order) derivatives of this curve, the extreme points in Figure 3a (corresponding to the zero points in Figure 3b) were calculated to separate the hydration process as different stages. Specifically, the initial pre-induction period was not analyzed here owing to the time consumption from adding water to the first measurement, and thus the subsequent four stages were identified including the induction (dormant) stage, acceleration stage, deceleration stage and steady stage.

Moreover, TAM Air isothermal calorimetry (TA Instruments, New Castle, DE, USA) was used to characterize the hydration kinetics of the cement paste samples by monitoring the heat release under a 20 °C constant temperature condition for parallel comparison.

## 3. Results and Discussion

### 3.1. Morphology and Compositions

**Figure 4:**
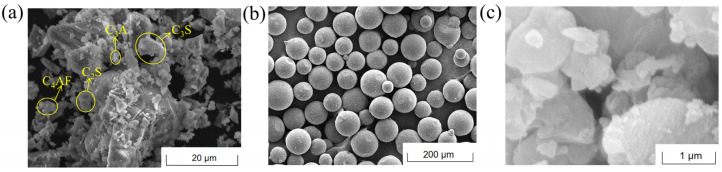
SEM observation images of raw materials: (**a**) cement; (**b**) SAP; (**c**) CaO-based EA.

The microstructure morphologies of the raw materials observed by the scanning electron microscope (SEM) are presented in Figure 4. The SEM micrographs revealed the mineralogical phases in Figure 4a, including angular C_3_S (tricalcium silicate, 10–50 μm, distinct cleavage planes), sub-rounded C_2_S (dicalcium silicate, 5–20 μm, surface etching), porous C_3_A aggregates (tricalcium aluminate, 2–10 μm), and equant C_4_AF crystals (tetracalcium ferroaluminate, 3–15 μm). The chemical compositions of the cement and CaO-based EA are shown in Table 2, which were analyzed by X-ray fluorescence (XRF) spectroscopy.

### 3.2. Comparison of LF-NMR and IC Results

**Figure 5:**
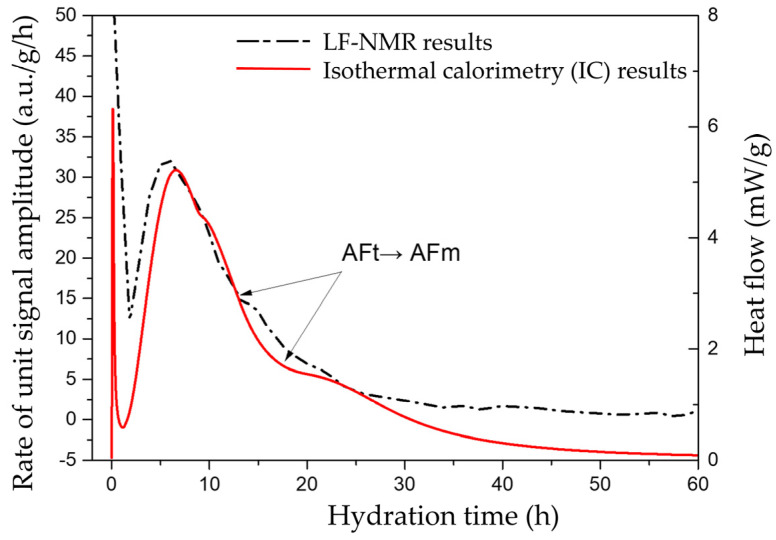
Comparative analysis of LF-NMR and isothermal calorimetry measurements for cement paste with *w*/*c* = 0.35.

The heat flow of the plain cement paste sample with a *w*/*c* of 0.35 being obtained by isothermal calorimetry and the first-order differential curve of the *T*_2_ signal maximum amplitude from LF-NMR characterization were collected and are compared in Figure 5. At a very early age of the hydration process, the reaction of C_3_A minerals first occurred once the solids contacted with water, after the dissolution of clinker minerals. The ettringite (AFt) hydrates formed rapidly to entry the pre-induction period, which was the main factor that resulted in the generation of the first exothermic peak. Afterward, the speed of the hydration reactions tended to slow down and enter into a short induction period, with the gradual accumulation of hydration products. In the later stage during the hydration for a further several hours, the second exothermic peak appeared with the intense formation of Ca(OH)_2_ and C-S-H gel products, indicating that a large amount of heat was released and thus the overall hydration kinetics were accelerated again. Finally, abundant hydration products filled the space that was occupied by water previously and then formed a network structure interlinked with hydration products, which decreased the contact area of the un-hydrated cement particles with water and thus led to the deceleration stage. A shoulder occurred in the NMR result curve at approximately 17 h, which could also be found in the heat flow curve at around 17 h, representing the transformation of AFt to calcium aluminate mono-sulfate (AFm). Ultimately, the hydration rate and exothermic rate tend to be stable. Thereby, the time-dependent hydration kinetics of the cement paste being indicated by LF-NMR showed a strong relation with the results obtained from traditional IC technique, which provide a convincing technique for monitoring the hydration process and investigating the role of CaO-based EA and SAP.

### 3.3. Influence of w/c on Hydration Stages

**Figure 6:**
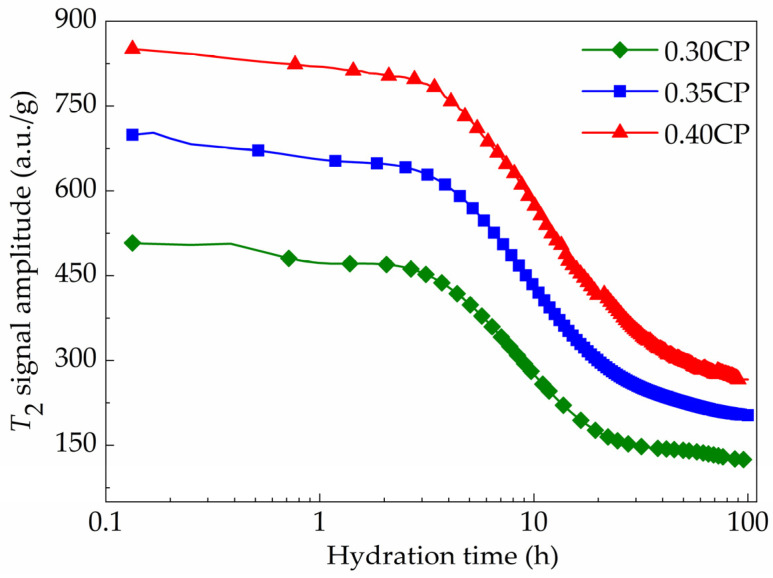
Time-dependent evolution of *T*_2_ signal amplitude for cement paste samples with variable *w*/*c* values.

**Figure 7:**
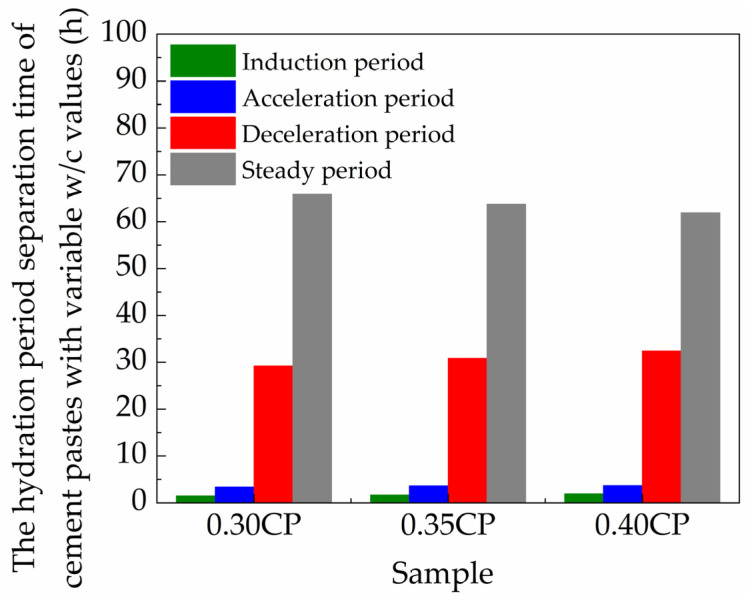
The duration of hydration stages for cement paste samples with variable *w*/*c* values.

The time-dependent evolution of the *T*_2_ signal maximum amplitude collected from the LF-NMR characterization is exhibited in Figure 6, which involves samples with three different *w*/*c* values (0.30, 0.35 or 0.40). Generally, it can be clearly observed that the intensity of the signal indicating the physically bound water showed similar decreasing tendencies with prolonged hydration time for all the samples. On the other hand, the *T*_2_ signal intensity became stronger with a higher *w*/*c* value, as expected, as the higher water content in the mixture means more physically bound water was contained by unit mass of cement paste. The influence of the mixture *w*/*c* value on each hydration stage was further analyzed, which was divided by the zero points in the second-order differential curve. The detailed parameters of every typical hydration period, including the initial stage, the acceleration stage, the deceleration stage and the steady stage for all samples, are provided in Table 3 and Figure 7.

During the induction stage, the *T*_2_ signal amplitude initially showed a rapid decreasing tendency. In the later portion of induction stage, a slower decreasing rate could be found for the *T*_2_ signal amplitude, owing to the formation of a protective membrane [32]. The early-age hydrates contributed to the generation of membranes being composed of C-S-H gel, covering the surface of the un-hydrated cement grains and impeding the successional permeation of cationic/anionic ions in the solution, which hindered the hydration during the later induction stage. In the acceleration stage, the decreasing rate of *T*_2_ signal amplitude became higher again with the supersaturation of calcium ions, and the promoted precipitation of Ca(OH)_2_ crystals consumed water rapidly in fresh cement paste mixture. These hydration reactions led to the fast-decreasing curve of *T*_2_ signal amplitude during this stage. At the terminal of the acceleration stage, the *T*_2_ signal intensities decreased to 402.3, 563.9 and 700.3 a.u./g for the 0.30CP, 0.35CP and 0.40CP samples, respectively. Taking a closer looking at the data, the average decline rates during the total acceleration stage were 20.3, 23.6 and 28.4 a.u./g/h for the 0.30CP, 0.35CP and 0.40CP samples, respectively. The results implied that water consumption by hydration reactions would be significantly more severe with a higher *w*/*c* of cement paste during the acceleration stage.

After the acceleration stage, the hydration of the cement paste entered in the deceleration stage as the generation of a large quantity of hydration products. At the end of deceleration stage, the *T*_2_ signal intensities further decreased to 146.2, 243.3 and 320.6 a.u./g for the 0.30CP, 0.35CP and 0.40CP samples, respectively. At the end of the deceleration stage, the reaction rate of hydration became quite slow, which manifested as the relative steady signal intensity, implying less variation in the physically bound water amount. The hydration time points for reaching the last steady hydration stage were about 34.1, 36.3 and 38.1 h for the 0.30CP, 0.35CP and 0.40CP samples, respectively. Hence, it took a longer time for the cement paste with a higher *w*/*c* ratio to achieve a stable hydration state. Additionally, it is worth noting that although the curves in Figure 6 exhibited slightly falling tendencies in the steady stage, the decreasing rates were substantially lower than before and could even actually be neglectable with the time prolonging because the horizontal axis for the time mark was on a logarithmic scale. Overall, a higher water content in the mixture resulted in the longer duration of every hydration stage, which may be explained by the higher dilution degree of the cementitious mixture causing a delayed hydration progress.

Zhao investigated the hydration process of Portland cement with *w*/*c* values of 0.3, 0.35 and 0.4. According to their results, with the increase in *w*/*c* from 0.3 to 0.35 or 0.4, the induction stage was prolonged by 6.2% or 9.7%, the acceleration time was prolonged by 2.9% or 6.4%, and the deceleration time was prolonged by 14.5% or 64.5%. The *T*_2_ signal amplitude per unit mass increased with a higher *w*/*c*. In the steady stage, the hydration reaction rate was extremely small, the content of physically bound water in cement paste was very little and hydration tended to become steady [33].

### 3.4. Influence of SAP on Hydration Stages

**Figure 8:**
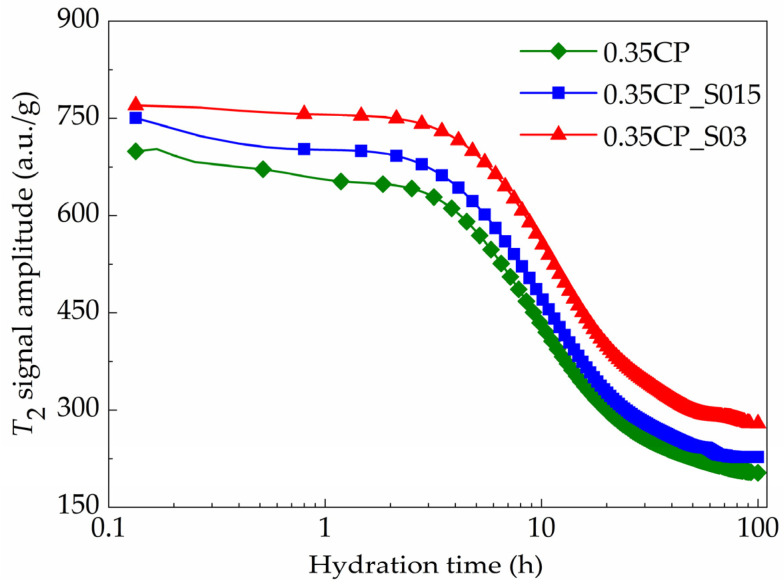
Time-dependent evolution of *T*_2_ signal amplitude for cement paste samples with variable SAP dosages (*w*/*c* = 0.35).

**Figure 9:**
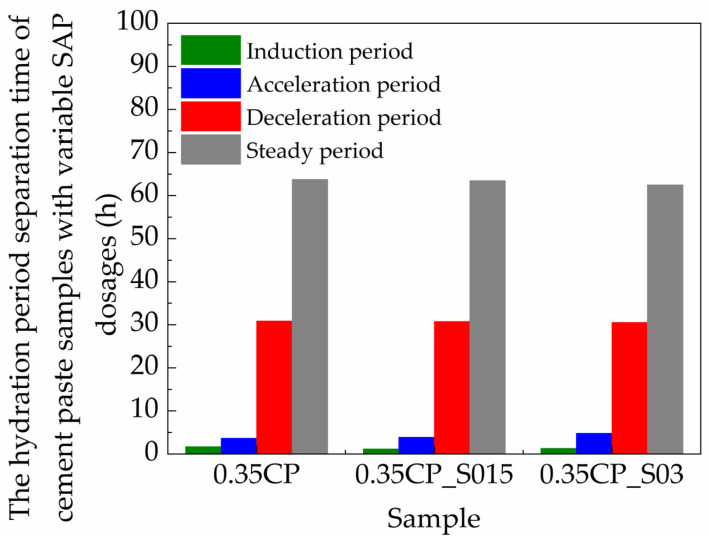
The duration of hydration stages for the cement paste samples with variable SAP dosages (*w*/*c* = 0.35).

The influence of the SAP content on the time-dependent evolution of the *T*_2_ signal maximum amplitude is presented in Figure 8. It can be clearly seen that the *T*_2_ signal intensity (denoting the amount of physically bound water) became stronger with the higher SAP dosage, which meant that the addition of the SAP could help to reserve the water content in the fresh cementitious mixture and reduce the water consumption amount during hydration reactions. However, the use of the SAP did not affect the overall trend of the *T*_2_ signal amplitude curve. The detailed parameters of the typical hydration periods involving cement paste samples with variable SAP dosages are provided in Table 4 and Figure 9. Then, the effect of the SAP on every specific hydration stage was further discussed accordingly.

Firstly, the duration of the induction stage continued for 1.72, 1.88 and 2.10 h for the 0.35CP, 0.35CP_S015 and 0.35CP_S03 samples, respectively. Hence, the incorporation of the SAP with a higher content prolonged the induction stage of cement hydration more evidently, owing to the increased total water amount in mixtures at the beginning. Meanwhile, after incorporating 0.15% or 0.30% SAP, the *T*_2_ signal intensity increased from 649.2 to 695.0 or 749.7 a.u./g at the end of induction period and subsequently delayed the onset of later exothermic peaks. Then, the acceleration stage of the 0.35CP, 0.35CP_S015 and 0.35CP_S03 samples continued for 3.64, 3.87 and 4.82 h, respectively. During this stage, the cement pastes gradually hardened and formed a dense skeleton, which could wrap the hydration products together with the un-hydrated cement grains. It is obvious that the higher SAP content prolonged the duration of the acceleration stage significantly, since the SAP particles started to continuously release water during this stage, which could be driven by the concentration gradient difference of ions in the liquid phase. In other words, the addition of the SAP was equivalent to the entrainment of extra water being effective for hydration reactions. This function of SAP particles would decrease the concentration of calcium ions in a pore solution, which may explain the extended duration of the acceleration stage. Such a retardation effect of the SAP on hydration was also in accordance with other previous results by isothermal calorimetry [18,19].

As for the subsequent deceleration stage, the differential calculation results showed that it terminated at 36.25, 36.53 and 37.47 h for the 0.35CP, 0.35CP_S015 and 0.35CP_S03 samples, respectively. So, the addition of a higher SAP content could slightly extend the ending time point of the deceleration stage, at which the *T*_2_ signal intensities were decreased from 243.3 to 265.7 and 323.1 a.u./g for the 0.35CP_S015 and 0.35CP_S03 samples, respectively. During this period, the SAP particles could release water gradually with the descending internal humidity in the cement paste and thus were able to endow the hardened materials with a sufficient and uniform distribution of interior moisture, which contributed to the promotion of the holistic hydration degree for the cement-based materials. Finally, the samples entered into the steady stage, and their curves presented horizontal extending trends, which suggested that the hydration reactions became much slower or even nearly static. The long-term signals also revealed that the quantity of physically free water in the hardened cement paste was enhanced by the incorporation of the SAP, which indicated that it can release water continuously for realizing a long-term internal curing effect, which would mitigate the self-desiccation of concrete and thus reduce the shrinkage and cracking risks [14]. After hydration for 100 h, the *T*_2_ signal amplitude values of the 0.35CP, 0.35CP_S015 and 0.35CP_S03 samples were 203.2, 227.4 and 279.3 a.u./g, respectively, at the ending time point of measurement.

Similarly, Zhang et al. employed isothermal calorimetry to investigate the influence of an SAP at dosages of 0.2%, 0.4%, 0.6% and 1.0% (by cement mass) on the early hydration kinetics of Portland cement (*w*/*c* = 0.5). The results demonstrated that the SAP modified the hydration process without altering the duration of the induction stage nor the timing of the acceleration stage onset. However, the SAP significantly increased the heat evolution rate during the acceleration period and elevated the main exothermic peak value compared to the control group without SAP addition [34].

### 3.5. Influence of CaO on Hydration Stages

**Figure 10:**
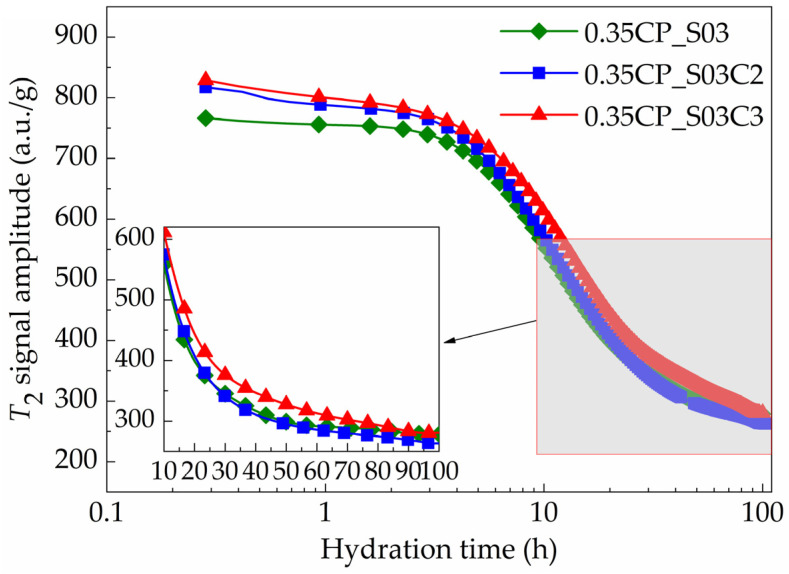
Time-dependent evolution of *T*_2_ signal amplitude for cement paste samples with constant SAP and variable CaO dosages (*w*/*c* = 0.35).

**Figure 11:**
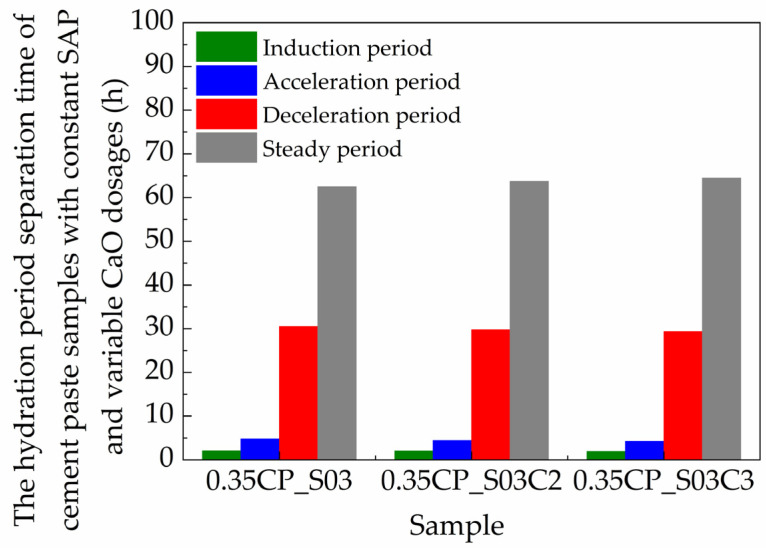
The duration of hydration stages for cement paste samples with constant SAP and variable CaO dosages (*w*/*c* = 0.35).

The use of the CaO-based EA may also exert significant influences on the hydration kinetics of the cement paste. This section focuses on the effect of the CaO-based EA with the dosages of 0, 2% and 3% and with the presence of the SAP as an internal curing admixture simultaneously. The time-dependent evolution of the *T*_2_ signal amplitude for these shrinkage-compensating cement pastes with different contents of the CaO-based EA are exhibited in Figure 10. Considering the fact that the hydration of cement usually requires a higher amount of water than that of a CaO-based EA with equivalent mass [35], the replacement of cement by more EA resulted in less water being consumed by hydration. Hence, when the *w*/*c* ratio was constant, using the CaO-based EA as a binder component to partially replace the cement may have facilitated the generation of more free water in early-age mixture. Correspondingly, the time portions for each hydration stage were further determined as listed in Table 5, and the division results are depicted in Figure 11.

For the induction stage, the incorporation of the CaO-based EA led to stronger *T*_2_ signal intensities, which was possibly because the addition of the CaO-based EA modified the particle-packing states of solids and thus released more free water from deflocculation for the fresh mixture initially [36]. At the end of the induction stage, the *T*_2_ signal maximum amplitudes of the 0.35CP_S03, 0.35CP_S03C2 and 0.35CP_S03C3 cement paste samples were 749.7, 777.3 and 787.5 a.u./g, respectively. On the other hand, plenty of calcium ions could be released by the dissolution of the CaO-based EA admixture, which may shorten the elapsed time being required for approaching its over-saturation concentration point [37]. So, the duration of the induction stage could be shortened with a higher content of the CaO-based EA. Then, when reaching the terminal time point of the acceleration hydration stage, the *T*_2_ signal amplitude intensities of the 0.35CP_S03, 0.35CP_S03C2 and 0.35CP_S03C3 samples were 641.4, 669.7 and 702.9 a.u./g, respectively. During this period, the corresponding average descending rates of the 0.35CP_S03 and 0.35CP_S03C2 samples were as high as 22.47 and 24.29 a.u./g/h, which indicated the promoted hydration reactions of cement clinker minerals to consume free or physically bound water and motivate the generation of hydration products more rapidly. In other words, it is interesting to note that the time-dependent signal curves exhibited a faster descending tendency when adding the CaO-based EA, probably because the dissolution of CaO can enhance the concentration of calcium ions in a solution [38]. Furthermore, it can be obviously found that the *T*_2_ signal amplitude curves of the 0.35CP_S03C2 and 0.35CP_S03C3 samples were rather close, with not very obvious variation between them if the dosage of the CaO-based EA increased from 2% to 3%.

Afterward, for the subsequent deceleration stage, the overall consumption rate of free water or physically bound water became obviously slower for all these mixtures. Specifically, the *T*_2_ signal amplitude values of the 0.35CP_S03 and 0.35CP_S03C2 samples decreased to 323.1 and 319.5 a.u./g at the end of deceleration stage, when the terminal time points were about 37.5 and 36.3 h from adding water. Thereby, the incorporation of the CaO-based EA shortened both the acceleration and deceleration stages, which verified the promotion effect of the CaO-based EA on the hydration kinetics of the cement paste with the presence of the SAP for internal curing. After about 20 h, the hardened cement pastes gradually entered into the steady stage. The amplitude values of the *T*_2_ signal fluctuated just around 278.3, 263.1 and 283.4 a.u./g for the 0.35CP_S03, 0.35CP_S03C2 and 0.35CP_S03C3 samples, respectively, after hydration for 100 h. In addition, regarding the 0.35CP_S03C2 sample, it consumed more water in the hydration reactions during the whole measurement period compared with the other two samples, as verified by the largest variation in *T*_2_ signal amplitude values at the beginning and ultimate moments. This indicated that 2 wt% could be a suitable dosage of CaO-based EA for reaching a more effective promotion effect on the hydration kinetics of cement paste.

Similarly, Zhao et al. used the LF-NMR technique to study the effect of a CaO-MgO blended EA on the early-age hydration of a cement paste with a *w*/*c* of 0.35. The results showed that with the increase in the CaO-MgO blended EA, the amount of transverse relaxation *T*_2_ signal in the induction period increased, and the induction stage was shortened by 16.0% and 20.3%, respectively, when the dosage of the CaO-MgO blended EA was increased from 0 to 2% and 4%. The use of the CaO-MgO blended EA was able to increase the concentration of Ca^2+^ in the pore solution of the cement slurry to shorten the duration of the induction period. However, with the increase in the CaO-MgO blended EA, the *T*_2_ signal intensity in the stable period decreased [39].

## 4. Research Significance

The experimental results provide valuable insights into advancing cement-based material technology. The identified hydration stages and their dependence on the *w*/*c* ratio offer a scientific basis for optimizing concrete mix designs, particularly in controlling setting times and strength development. The SAP addition strategy demonstrates potential for developing internal curing concrete systems, which could significantly improve durability in dry climates or water-scarce regions. Furthermore, the synergistic effects of the SAP and CaO-based EA open new possibilities for designing shrinkage-compensated concrete, addressing a critical challenge in modern construction. These findings lay a solid foundation for developing high-performance concrete with enhanced performance and sustainability. In the future, more in-depth investigations will be carried out to further explore the coupled effects of SAPs and CaO on the volume deformation and cracking tendency of early-age cement-based materials, based on the hydration analysis results in this study.

## 5. Conclusions

According to the experimental investigations, some major conclusions can be obtained as follows:

(1) For fresh cement paste samples, the time-dependent evolution of transverse relaxation time (*T*_2_) signal maximum amplitude presented a descending tendency with prolonged hydration time. Based on the extreme points in the first-order derivatives of the *T*_2_ signal maximum amplitude curve, the hydration progress could be further identified with four different stages.

(2) With the increase in the water-to-cement ratio (*w*/*c*) in the cementitious mixture, all the stages in hydration progress (including induction, acceleration, deceleration and steady stages) became longer, which could be explained by more physically bound or free water in the mixture that led to a higher dilution degree.

(3) The incorporation of the super-absorbent polymer (SAP) with a higher content could retard the hydration kinetics more evidently at a very early age and thus prolong the duration of the induction and acceleration stages. Afterwards, the SAP also exhibited an effective promotion on the overall hydration degree of the cement-based materials at later stages.

(4) Adding the CaO-based expansive admixture (EA) shortened the induction, acceleration and deceleration stages of the cement paste, which confirmed its promotion effect on hydration kinetics with the presence of the SAP. The combination of 3 wt% SAP and 2 wt% CaO could consume more water synergistically by hydration reactions in the first 100 h.

## Figures and Tables

**Table 1:** Mix proportions of cement paste samples.

Sample	*w*/*c*	SAP (%)	EA (%)	Extra Water for SAP Absorption (%)
0.30CP	0.30	0	0	0
0.40CP	0.40	0	0	0
0.35CP	0.35	0	0	0
0.35CP_S015	0.35	0.15	0	2.96
0.35CP_S03	0.35	0.30	0	5.91
0.35CP_S03C2	0.35	0.30	2.0	5.91
0.35CP_S03C3	0.35	0.30	3.0	5.91

**Table 2:** Chemical compositions of cement and CaO-based EA (wt%).

Material	CaO	SiO_2_	Al_2_O_3_	Fe_2_O_3_	MgO	K_2_O	Na_2_O	TiO_2_	SO_3_	LOI
Cement	64.77	19.62	4.53	3.15	0.89	0.64	0.07	0.26	2.40	1.67
EA	84.32	3.38	4.93	1.20	1.26	—	—	—	2.57	2.34

**Table 3:** The starting and terminal time points of each hydration stage for cement paste samples with variable *w*/*c* values.

Sample	Hydration Time (Hours:Minutes)			
	Induction Stage	Acceleration Stage	Deceleration Stage	Steady Stage
0.30CP	[0*,1:31]	[1:31,4:55]	[4:55,34:08]	[34:08,100*]
0.35CP	[0*,1:43]	[1:43,5:22]	[5:22,36:15]	[36:15,100*]
0.40CP	[0*,1:58]	[1:58,5:40]	[5:40,38:05]	[38:05,100*]

Note: The 0* indicates the time point at which the first measurement was taken but not the cement coming into contact with water. The 100* indicates the end of the measurement.

**Table 4:** The starting and terminal time points of each hydration stage for cement paste samples with variable SAP dosages (*w*/*c* = 0.35).

Sample	Hydration Time (Hours:Minutes)			
	Induction Stage	Acceleration Stage	Deceleration Stage	Steady Stage
0.35CP	[0*,1:43]	[1:43,5:22]	[5:22,36:15]	[36:15,100*]
0.35CP_S015	[0*,1:53]	[1:53,5:45]	[5:45,36:32]	[36:32,100*]
0.35CP_S03	[0*,2:06]	[2:06,6:55]	[6:55,37:28]	[37:28,100*]

Note: The 0* indicates the time point at which the first measurement was taken but not the cement coming into contact with water. The 100* indicates the end of the measurement.

**Table 5:** The starting and terminal time points of each hydration stage for cement paste samples with constant SAP and variable CaO dosages (h).

Sample	Hydration Time (Hours:Minutes)			
	Induction Stage	Acceleration Stage	Deceleration Stage	Steady Stage
0.35CP_S03	[0*,2:06]	[2.:06,6:55]	[6:55,37:28]	[37:28,100*]
0.35CP_S03C2	[0*,2:04]	[2:04,6:29]	[6:29,36:17]	[36:17,100*]
0.35CP_S03C3	[0*,1:56]	[1:56,6:11]	[6:11,35:32]	[35:32,100*]

Note: The 0* indicates the time point at which the first measurement was taken but not the cement coming into contact with water. The 100* indicates the end of the measurement.

## Data Availability

The original contributions presented in the study are included in the article, further inquiries can be directed to the corresponding author.
